# Feasibility and reliability of fetal two dimensional speckle tracking echocardiography at 16 weeks gestational age: A pilot study

**DOI:** 10.1371/journal.pone.0302123

**Published:** 2024-04-17

**Authors:** Thomas J. Nichting, Zoé A. van Lier, Chantelle de Vet, Myrthe van der Ven, Daisy A. A. van der Woude, Sally A. Clur, Noortje H. M. van Oostrum, S. Guid Oei, Judith O. E. H. van Laar

**Affiliations:** 1 Department of Gynaecology and Obstetrics, Máxima MC, Veldhoven, The Netherlands; 2 Department of Electrical Engineering, Eindhoven University of Technology, Eindhoven, The Netherlands; 3 Eindhoven MedTech Innovation Centre, Eindhoven, The Netherlands; 4 Faculty of Health, Medicine and Life Sciences, Maastricht University, Maastricht, The Netherlands; 5 Department of Biomedical Engineering, Eindhoven University of Technology, Eindhoven, The Netherlands; 6 Department of Pediatric Cardiology, Emma Children’s Hospital, Amsterdam University Medical Centers, Amsterdam, The Netherlands; 7 Member of the European Reference Network for Rare, Low Prevalence and Complex Diseases of the Heart–ERN GUARD-Heart, Amsterdam, The Netherlands; 8 Department of Gynaecology and Obstetrics, University Hospital Gent, Gent, Belgium; Sun Yat-Sen University, CHINA

## Abstract

**Background:**

Fetal two-dimensional speckle tracking echocardiography (2D-STE) is an emerging technique for assessing fetal cardiac function by measuring global longitudinal strain. Alterations in global longitudinal strain may serve as early indicator of pregnancy complications, making 2D-STE a potentially valuable tool for early detection. Early detection can facilitate timely interventions to reduce fetal and maternal morbidity and mortality. Therefore, the aim of this study was to investigate the feasibility of performing 2D-STE at 16 weeks gestational age.

**Methods:**

This pilot study utilized 50 ultrasound clips of the fetal four-chamber view recorded between 15+5 and 16+2 weeks gestational age from a prospective cohort study. A strict protocol assessed three parameters essential for 2D-STE analysis: fetal four-chamber view ultrasound clip quality, region of interest, and frame rates. Two independent researchers measured global longitudinal strain in all adequate fetal four-chamber view ultrasound clips to determine inter- and intra-operator reliability.

**Results:**

Out of the 50 ultrasound clips, 37 (74%) were feasible for 2D-STE analysis. The inter-operator reliability for global longitudinal strain measurements of the left and right ventricles was moderate (ICC of 0.64 and 0.74, respectively), while the intra-operator reliability was good (ICC of 0.76 and 0.79, respectively).

**Conclusions:**

Our findings demonstrate that fetal 2D-STE analysis at 16 weeks gestational age is feasible when adhering to a strict protocol. However, further improvements are necessary to enhance the inter- and intra-operator reliability of 2D-STE at this gestational age.

## Introduction

Early detection of a fetus at risk due to abnormal placentation is vital. It enables adjustments in prenatal care, as well as decisions regarding treatment or the timing and location of delivery. Abnormal placentation is the common pathophysiological pathway in fetal growth restriction and hypertensive pregnancy disorders. A higher resistance of the placental bed results in an increased afterload [1, 2]. In order to maintain cardiac output, the fetal heart starts to remodel, transitioning to a more globular shape and eventually progressing towards hypertrophy. The current, indirect, approach for identifying indications of impaired placentation relies on Doppler measurements of the umbilical artery. Nevertheless, Doppler measurements tend to become abnormal relatively late, when already between 60 to 70 percent of the placental vessels are irreversibly damaged [3, 4].

Fetal two-dimensional speckle tracking echocardiography (2D-STE) is an emerging technique used to directly assess remodeling changes. This technique allows for the measurement of fetal cardiac deformation during one cardiac cycle, specifically using global longitudinal strain (GLS), which is considered the most useful and reliable strain parameter in fetuses [5], [6]. Abnormal GLS values have been observed in various pregnancy complications, such as fetal growth restriction and hypertensive pregnancy disorders [7, 8]. Increased GLS values have been suggested as the earliest sign of fetal growth restriction [9]. Therefore, 2D-STE holds promise as a technique for the early detection of pregnancy complications.

In 2D-STE, the displacement of speckles, which are reflections of the ultrasound beam by the fetal myocardium, is tracked. These speckles can be monitored frame to frame throughout a cardiac cycle [10]. To accurately track the speckles and measure GLS, it is crucial to adhere to a strict protocol for obtaining adequate ultrasound clips of the fetal four-chamber view (4CV) [6]. The requirements for optimal imaging include acquiring a high-quality 4CV ultrasound clip that fills most of the ultrasound screen. This can be achieved by using optimal settings for the region of interest (ROI) and high framerates, including frames per second (FPS) and frames per cardiac cycle (FPC) [6, 11–13]. However, the smaller size of the fetal heart, higher heart rate, and increased fetal movements compared to later gestational periods may pose challenges in obtaining adequate 4CV ultrasound clips for 2D-STE analysis [5, 14].

If feasible at early gestational age, timely intervention may aid in reducing perinatal morbidity and mortality, such as by reducing the risks of abnormal placentation by initiating preventive treatment with low-dose acetylsalicylic acid. For optimal effectiveness, treatment should start before the end of the 16th week of pregnancy [15].

Studies on 2D-STE feasibility have primarily focused on gestational periods between 18 and 41 weeks, and reference values have only been established for these periods [16, 17]. Limited research exists on the feasibility of 2D-STE before the 16th week of pregnancy, with one study reporting a success rate of only 36% for 2D-STE in fetuses between 12 and 14 weeks of (GA) [13].

The objective of this study is to assess the feasibility of fetal 2D-STE around 16 weeks GA and evaluate the inter- and intra-operator reliability of the technique.

## Materials and methods

### Study design

This pilot study was conducted as part of a larger longitudinal prospective cohort study, the BEATS study. The study protocol for the BEATS study has been previously published [18]. The research was approved by the medical ethics committee of the Máxima Medical Center in Veldhoven, the Netherlands (NL73607.015.20). Within the BEATS study, ultrasound clips of the 4CV were acquired every four weeks at 16, 20, 24, and 28 weeks of GA. For this pilot study, we analyzed the first 50 4CV ultrasound clips obtained at 16 weeks GA. These clips were collected between November 2021 and July 2022.

### Study population

Women were enrolled in the study during their initial appointment before reaching 16 weeks of GA at either a tertiary care hospital or a primary midwifery clinic in The Netherlands. Prior to participation, all women provided both verbal and written informed consent. Eligibility criteria included healthy women with a low-risk singleton pregnancy. Exclusion criteria consisted of women with pre-existing maternal diseases that could potentially impact fetal cardiac development, such as diabetes mellitus or pre-existing hypertensive disease, as well as cases where the fetus had been diagnosed with a cardiac abnormality (e.g. cardiac arrhythmias or congenital cardiac anomalies). To be included in the study, a fetal heart clip had to be obtained between 15+5 and 16+2 weeks of GA.

### Data acquisition

The ultrasound system used for this study was the Philips Epiq W7 (Royal Philips N.V., Amsterdam, The Netherlands), which was equipped with a 9-MHz linear transducer. To minimize artifacts caused by maternal movements, pregnant women were instructed to hold their breath during the acquisition of the 4CV ultrasound clips [18]. Each 4CV ultrasound clip had a duration of 3 seconds and captured multiple cardiac cycles. The raw, uncompressed data from the fetal heart clips was used for offline analysis using the TomTec 2D Cardiac Performance 1.2 software (TomTec Imaging Systems GmbH, Munich, Germany). The use of raw data ensured that frame rates were not compressed during storage of the 4CV ultrasound clips [19].

### Feasibility assessment

Feasibility was evaluated according to a strict protocol designed to ensure reliable 2D-STE analysis. The assessment process involved multiple steps. Firstly, the quality of the 4CV ultrasound clip was examined. Secondly, the ROI setting was evaluated. Lastly, the frame rates were reviewed, aiming for a high enough rate without compromising the quality of the ultrasound clip [6, 20]. For the 4CV ultrasound clip to be considered feasible for 2D-STE analysis, all three parameters needed to meet the predefined criteria. Further details regarding these three parameters are described below and summarized in Table 1.

**Fig 1:**
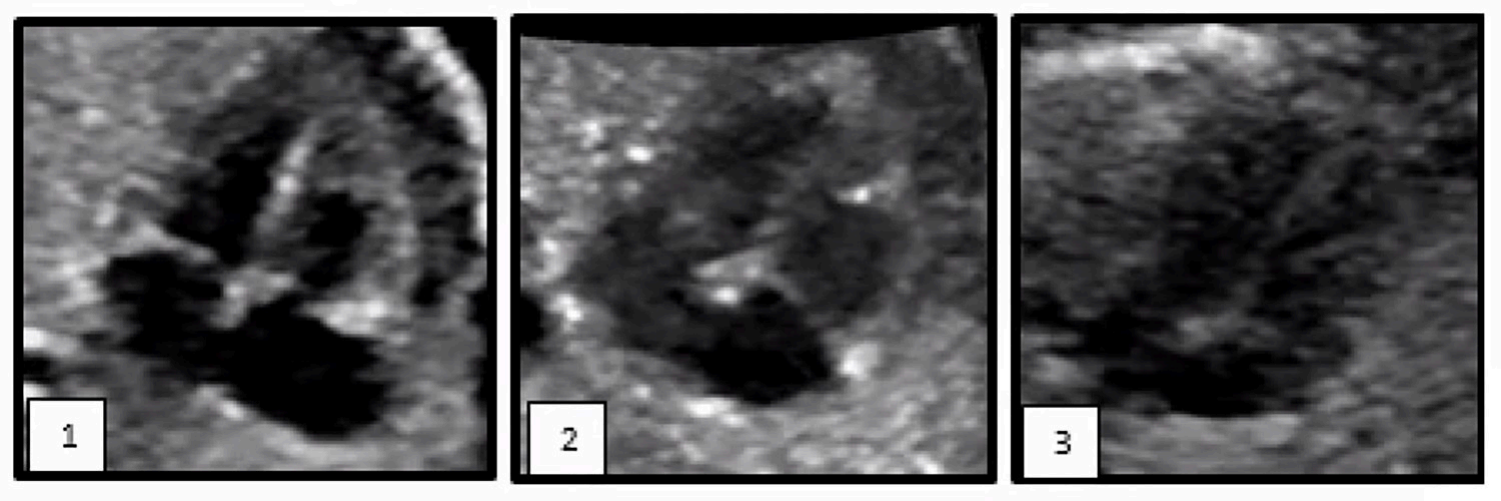
Evaluation of quality of four-chamber view ultrasound clips.

1. We categorized *the quality of the 4CV ultrasound clips* as either good, moderate, or low quality [36]. Good quality 4CV ultrasound clips exhibited a complete four-chamber view, clearly defining the boundaries between the endocardium, lumen, and atrioventricular valves. In moderate and low quality 4CV ultrasound clips, there was limited or no clear distinction between the endocardium, lumen, and atrioventricular valves. Moreover, low quality 4CV ultrasound clips may have been affected by acoustic shadows, obscuring cardiac structures and preventing accurate delineation. Ultrasound clips of good and moderate quality were deemed adequate for 2D-STE analysis [6]. Fig 1 provides visual examples of good, moderate, and low quality 4CV ultrasound clips.
2. We classified *the ROI* for each clip as either optimal, suboptimal, or bad. An optimal ROI was achieved when the sector width was narrow, the imaging depth was shallow, and the zoom box was utilized effectively, resulting in a 4CV ultrasound clip that occupied the entire screen. A suboptimal ROI was characterized by the 4CV ultrasound clip filling ≥50% of the ultrasound clip, despite the presence of other fetal structures. The ROI was considered bad when the 4CV ultrasound clip filled <50% of the ultrasound clip. Both an optimal and suboptimal ROI were considered adequate for 2D-STE analysis.
3. *The FPS* was required to be 80 frames per second or higher [5, 6]. Additionally, to account for the elevated fetal heart rate, FPC needed to reach 40 frames per cardiac cycle or higher for the clips to be deemed adequate [5, 6].

**Table 1:** Parameters used to assess feasibility.

*Parameter*	*Feasible*	*Not feasible*
4CV ultrasound clip quality	Moderate or high quality	Low quality
ROI	Optimal or suboptimal	Bad
Frame rates		
FPS	≥ 80	< 80
FPC	≥ 40	<40

**4CV**: fetal four-chamber view, **ROI:** region of interest, **FPS**: frames per second, **FPC**: frames per cardiac cycle.

1. Example of a good quality four-chamber view ultrasound clip, clearly defining the boundaries between the endocardium, lumen, and atrioventricular valves.
2. Example of a moderate quality four-chamber view ultrasound clip, in which distinction between the endocardium, lumen, and atrioventricular valves is limited.
3. Example of a low quality four-chamber view ultrasound clip were no distinction is possible. In low quality 4CV ultrasound clips acoustic shadows may hinder accurate delineation.

The fetal 4CV ultrasound clips were assessed by two researchers (TN and ZvL). When disagreement occurred, it was resolved through discussion between the researchers. If a consensus could not be reached, an independent researcher (NvO) with more than ten years of experience in fetal echocardiography and over five years of experience in fetal 2D-STE analysis was consulted.

### Reliability

The inter- and intra-operator reliability was calculated. GLS measurements were solely performed on 4CV ultrasound clips that met the feasibility criteria. Both researchers (TN, ZvL) independently performed all GLS measurements, blinded to each other’s results. Additionally, one researcher (ZvL) repeated the GLS measurements after a minimum interval of one week, while remaining blinded to her initial GLS measurements.

### 2D-STE analysis

GLS refers to the percentage change in myocardial length from end-diastole to end-systole within one cardiac cycle and is expressed as a negative value. A greater shortening of the myocardium (i.e., a more negative GLS value) was defined as a mathematically reduced GLS value [21]. Both left ventricular (LV) GLS and right ventricular (RV) GLS were calculated. To measure GLS, the following five steps were performed using the offline 2D-STE software [6].

1. The M-mode feature was utilized to isolate a single cardiac cycle;
2. In the end-systolic frame, three reference points were positioned to locate the fetal heart’s apex and the lateral and septal walls at the annulus level of the mitral valve. The Quiver tool was employed to assist in identifying the reference points [22];
3. The software automatically positioned tracking lines along the endocardial border in the end-systolic frame. If necessary, manual adjustments were made to the tracking lines. The 2D-STE software utilized the narrowest width of the endocardial wall layer as the default for analysis [12];
4. Steps 2 and 3 were repeated for the end-diastolic frame;
5. GLS was calculated by the 2D-STE software.

### Sample size

According to the literature, pilot studies are recommended to have sample sizes ranging from 12 to 70 subjects [23]. Additionally, it is suggested that the minimum sample size for a pilot study should be 9% of the intended sample size for the main trial [23]. Hence, a total of 50 participants, approximately 9% of the intended subjects in the main trial analysis, were included in this pilot study [18].

### Statistical analysis

The data was analyzed using SPSS version 22 (The International Business Machines Corporation, Armonk, United States). Normality of distribution was assessed through histograms and the Shapiro-Wilk test. Continuous variables were reported as means and standard deviations (±SD) or medians and interquartile ranges (IQR) as appropriate. Categorical variables were presented as absolute numbers and percentages.

Feasibility was determined by examining the success percentage and the 95% confidence interval (95% CI) with continuity correction. To consider any potential deviation between the actual and projected results, a margin of error was calculated [24].

Inter- and intra-operator reliability were analyzed using the intra-class correlation coefficient (ICC) and Bland-Altman scatter plots. The ICC was calculated using a two-way mixed effects model with an absolute agreement definition. An ICC value below 0.50, between 0.50 and 0.75, between 0.75 and 0.90, and above 0.90 were indicative of poor, moderate, good, and excellent reliability, respectively [25].

## Results

**Fig 2:**
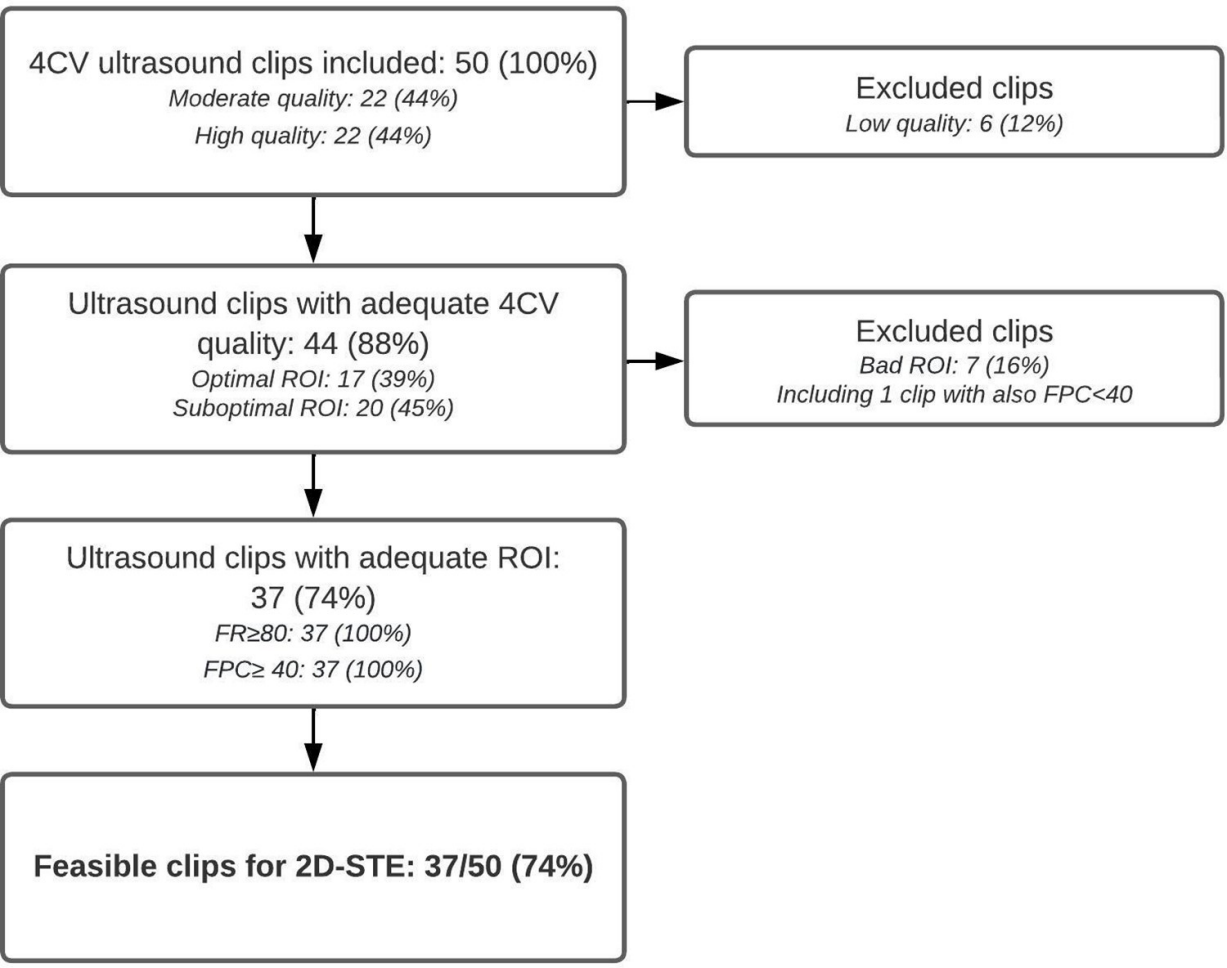
Flowchart of feasibility assessment.

Fig 2 shows the flowchart of the assessment of the 4CV ultrasound clips in order to asses feasibility. Fifty 4CV ultrasound clips were included for this pilot study. Of the 50 4CV ultrasound clips, 37 (74%) were feasible to perform 2D-STE analysis (95% CI 0.59–0.85) with a corresponding margin of error of 0.12.

Of the 13 (26%) 4CV ultrasound clips considered to be inadequate for 2D-STE analysis, six had a low 4CV ultrasound clip quality and seven had a bad ROI. One of these also had a FPC below 40 frames per cardiac cycle.

### Inter- and intra-operator reliability

Mean GLS values and the ICC results are shown in Table 2. The inter-operator reliability was found to be moderate and the intra-operator reliability was found to be good for both LV-GLS and RV-GLS. The p-values for the inter-operator ICC and intra-operator ICC were not statistically significant.

**Table 2:** The inter- and intra-operator reproducibility.

	*Operator 1 (SD)*	*Repeat operator 1 (SD)*	*Operator 2 (SD)*	*Inter-operator ICC [95% CI]*	*p-value*	*Intra-operator ICC [95% CI]*	*p-value*
LV-GLS	-19.85 (5.32)	-18.87 (4.92)	-19.41 (5.57)	0.67 [0.45–0.82]	0.62	0.75 [0.56–0.86]	0.11
RV-GLS	-21.33 (6.72)	-21.01 (5.52)	-20.65 (4.63)	0.75 [0.57–0.86]	0.46	0.80 [0.64–0.89]	0.82

## Discussion

This prospective study aimed to assess the feasibility and reliability of 2D-STE analysis at 16 weeks GA. A development that holds the potential for early detection of pregnancy complications and the subsequent reduction of fetal and maternal morbidity and mortality through timely intervention [26, 27]. Feasibility was evaluated using a strict protocol with three essential parameters for adequate 4CV ultrasound clips for 2D-STE analysis. Out of the 50 included clips, 37 (74%, 95% CI: 0.59–0.85) were deemed feasible for 2D-STE analyses. The inter-operator reliability for LV-GLS and RV-GLS measurements was moderate (ICC of 0.67 and 0.75, respectively), while the intra-operator reliability was good (ICC of 0.75 for LV-GLS and 0.80 for RV-GLS).

To date, only one study has investigated the feasibility of fetal GLS measurements between 12 and 14 weeks GA [13]. The findings of this retrospective study indicate that performing 2D-STE analysis within this GA range is feasible, with a success percentage of 36% (95% CI: 23–50%). In our pilot study conducted at 16 weeks GA, the feasibility of 2D-STE analysis increased significantly, with a success percentage of 74% (95% CI: 59–85%).

It might be due to their study design that the feasibility of 2D-STE at 12 to 14 weeks is lower compared to our pilot study. The ultrasound clips utilized in the study of Chelliah et al. underwent retrospective assessment, drawn from clinically indicated first-trimester ultrasounds. These clips, retrospectively analyzed, were not originally intended for the specific purpose of assessing 2D-STE feasibility. Consequently, it is possible that the sonographer did not consider all the necessary components crucial for conducting 2D-STE. This resulted in 17 out of the 53 fetuses having excessive fetal motion or inadequately visualized fetal heart clips, particularly those encompassing the outflow tracts, rendering them infeasible for analysis [13].

It is important to note that the inter- and intra-operator reliability of GLS measurements at 16 weeks GA is still lower compared to the reliability reported during later gestational periods [13–14, 21, 28–33]. Previous literature on inter- and intra-operator reliability has shown ICC values indicating good to excellent reliability between 18 and 41 weeks GA [32]. Feasibility have been assessed and was proven feasible in gestational periods around 20 weeks gestational age with a success percentage of circa 94 percent [34, 35]. In contrast, our pilot study at 16 weeks GA revealed moderate to good reliability. This suggests that accurately placing the tracking lines manually is more challenging at 16 weeks GA, even when utilizing 4CV ultrasound clips that are deemed feasible for 2D-STE measurements.

The increased acquisition of feasible 4CV ultrasound clips for 2D-STE analysis at 16 weeks GA compared to 12 to 14 weeks, as well as the decreased reliability at 16 weeks GA compared to later stages of pregnancy, can be attributed to the exponential growth of the fetal heart. During this exponential growth phase, the fetal heart undergoes significant development and becomes larger [36]. This increased size of the fetal heart allows for a clearer distinction between the various cardiac structures, facilitating more accurate placement of the tracking lines required for 2D-STE analysis [37]. Consequently, at a later GA, when the heart is more developed and larger, the reliability of GLS measurements tends to improve due to better visualization and delineation of cardiac structures.

Another explanation for the increased feasibility during later GA periods could be attributed to the changing characteristics and frequency of fetal movement as GA progresses. As the autonomic nervous system matures, there is a higher occurrence and longer periods of fetal rest [38]. This increase in rest periods makes it easier to properly set the ROI before the position of the fetal heart changes and becomes difficult to capture the heart within the ultrasound clip.

Within this study, it was necessary to exclude seven 4CV ultrasound clips due to a bad ROI. In each of these cases, the exclusion was a direct consequence of the presence of fast and frequent fetal movements, which posed challenges for the sonographer in obtaining a better ROI within the maximum allotted time frame of 30 minutes to acquire the 4CV ultrasound clip. Nevertheless, in order to enhance the clinical applicability, a moderate ROI was deemed adequate as long as the FPS and FPC were high and the quality of the 4CV ultrasound clip met the necessary standards [20, 38].

### Strengths and limitations

This is the first study representing the feasibility of 2D-STE at 16 weeks GA. The utilization of a strict protocol in this study may have contributed to the higher levels of feasible ultrasound clips and reliability observed compared to existing literature. Nonetheless, it is important to acknowledge that, being a pilot study, the study’s sample size was relatively small, which limits the generalizability of the findings. Additionally, the definitions utilized for the three parameters used to assess feasibility have not been previously validated, although they were carefully derived and compiled based on guidelines and studies pertaining to fetal 2D-STE. Finally, it is important to highlight that the angle of insonation (AoI) was not taken into account as a parameter when assessing feasibility. It has always been acknowledged that 2D-STE is independent of the AoI [5, 6, 11, 22, 39]. However, recent literature has raised concerns about the potential impact, suggesting that, particularly the up-down AoI, may result in significant differences in GLS values [11, 40]. Nevertheless, it is important to mention that this study did not include measurements of the up-down AoI and that this omission was unintentional and occurred by chance.

Further studies should be conducted to validate the findings of this pilot study. The implementation of a strict protocol in this study increased the feasibility of 2D-STE at 16 weeks GA to 74 percent compared to 36 percent at 12 to 14 weeks GA [13]. However, it is evident that the reliability of 2D-STE at 16 weeks GA lags behind when compared to later gestational periods. To enhance the reliability of 2D-STE and advance research in this field, we recommend the development of internationally recognized guidelines aiming to optimize the feasibility and reliability of the technique. Additionally, if high feasibility is consistently observed in subsequent studies, it would be valuable to establish reference values to explore the clinical applications of 2D-STE at 16 weeks GA.

## Conclusion

Acquiring adequate 4CV ultrasound clips at 16 weeks GA for performing 2D-STE measurements is feasible. However, there is a need for further enhancements in the intra- and inter-reliability of 2D-STE.

## Abbreviations

AoI: Angle of Insonation
FPC: Frames Per Cardiac Cycle
FPS: Frames Per Second
GA: Gestational Age
GLS: Global Longitudinal Strain
ICC: Intra-Class Correlation Coefficient
IQR: Inter Quartile Range
ROI: Region Of Interest
SD: Standard Deviation
2D-STE: Two-dimensional Speckle Tracking Echocardiography
4CV: Fetal Four-Chamber View
95%CI: 95 Percent Confidence Interval

## References

[1] Salavati N, Smies M, Ganzevoort W, Charles AK, Erwich JJ, Plösch T, et al. The Possible Role of Placental Morphometry in the Detection of Fetal Growth Restriction. Front Physiol. 2019 Jan 8;9:1884. doi: 10.3389/fphys.2018.01884 ; PMCID: PMC6331677. 30670983 PMC6331677

[2] Folk DM. Hypertensive Disorders of Pregnancy: Overview and Current Recommendations. J Midwifery Womens Health. 2018 May;63(3):289–300. doi: 10.1111/jmwh.12725 Epub 2018 May 15. . 29764001

[3] Thompson RS, Trudinger BJ. Doppler waveform pulsatility index and resistance, pressure and flow in the umbilical placental circulation: an investigation using a mathematical model. Ultrasound Med Biol. 1990;16(5):449–58. doi: 10.1016/0301-5629(90)90167-b . 2238251

[4] Alfirevic Z, Stampalija T, Dowswell T. Fetal and umbilical Doppler ultrasound in high-risk pregnancies. Cochrane Database Syst Rev. 2017 Jun 13;6(6):CD007529. doi: 10.1002/14651858.CD007529.pub4 ; PMCID: PMC6481396. 28613398 PMC6481396

[5] Germanakis I, Gardiner H. Assessment of fetal myocardial deformation using speckle tracking techniques. Fetal Diagn Ther. 2012;32(1–2):39–46. doi: 10.1159/000330378 Epub 2012 May 24. . 22626849

[6] DeVore GR, Polanco B, Satou G, Sklansky M. Two-Dimensional Speckle Tracking of the Fetal Heart: A Practical Step-by-Step Approach for the Fetal Sonologist. J Ultrasound Med. 2016 Aug;35(8):1765–81. doi: 10.7863/ultra.15.08060 Epub 2016 Jun 27. . 27353066

[7] Semmler J, Garcia-Gonzalez C, Sanchez Sierra A, Gallardo Arozena M, Nicolaides KH, Charakida M. Fetal cardiac function at 35–37 weeks’ gestation in pregnancies that subsequently develop pre-eclampsia. Ultrasound Obstet Gynecol 2021;57:417–22. doi: 10.1002/uog.23521 33098138

[8] van Oostrum NHM, Derks K, van der Woude DAA, Clur SA, Oei SG, van Laar JOEH. Two-dimensional Speckle tracking echocardiography in Fetal Growth Restriction: a systematic review. Eur J Obstet Gynecol Reprod Biol. 2020 Nov;254:87–94. doi: 10.1016/j.ejogrb.2020.08.052 Epub 2020 Sep 2. . 32950891

[9] van Oostrum NHM, van der Woude DAA, Clur SB, Oei SG, van Laar JOEH. Right ventricular dysfunction identified by abnormal strain values precedes evident growth restriction in small for gestational age fetuses. Prenat Diagn. 2020 Dec;40(12):1525–1531. doi: 10.1002/pd.5805 Epub 2020 Aug 16. . 32735353

[10] Blessberger H, Binder T: Non-invasive imaging: Two dimensional speckle tracking echocardiography: Basic principles. *Heart* 2010; 96: 716– 722. doi: 10.1136/hrt.2007.141002 20424157

[11] Semmler J, Day TG, Georgiopoulos G, Garcia-Gonzalez C, Aguilera J, Vigneswaran TV et al., Fetal Speckle-Tracking: Impact of Angle of Insonation and Frame Rate on Global Longitudinal Strain. J Am Soc Echocardiogr. 2020 Sep;33(9):1141–1146.e2. doi: 10.1016/j.echo.2020.03.013 Epub 2020 May 15. . 32423727

[12] Omdal TR, Khan U, Ebbing C, Kessler J, Karlsen HO, Leirgul E et al., The influence of region of interest width in fetal 2D-speckle tracking echocardiography late in pregnancy. Int J Cardiovasc Imaging. 2021 Nov 3. doi: 10.1007/s10554-021-02455-1 Epub ahead of print. . 34734368 PMC11129966

[13] Chelliah A, Dham N, Frank LH, Donofrio M, Krishnan A. Myocardial strain can be measured from first trimester fetal echocardiography using velocity vector imaging. Prenat Diagn. 2016 May;36(5):483–8. doi: 10.1002/pd.4813 Epub 2016 Apr 7. 26991266

[14] Kapusta L, Mainzer G, Weiner Z, Deutsch L, Khoury A, Haddad S et al., Changes in fetal left and right ventricular strain mechanics during normal pregnancy. J Am Soc Echocardiogr. 2013 Oct;26(10):1193–200. doi: 10.1016/j.echo.2013.06.007 23880053

[15] Cui Y, Zhu B, Zheng F. Low-dose aspirin at ≤16 weeks of gestation for preventing preeclampsia and its maternal and neonatal adverse outcomes: A systematic review and meta-analysis. Exp Ther Med. 2018 May;15(5):4361–4369. doi: 10.3892/etm.2018.5972 Epub 2018 Mar 20. ; PMCID: PMC5920352. 29725376 PMC5920352

[16] Lee-Tannock A, Hay K, Gooi A, Kumar S. Global longitudinal reference ranges for fetal myocardial deformation in the second half of pregnancy. J Clin Ultrasound. 2020 Sep;48(7):396–404. doi: 10.1002/jcu.22826 Epub 2020 Mar 19. . 32191357

[17] van Oostrum NHM, de Vet CM, Clur SB, van der Woude DAA, van den Heuvel ER, Oei SG et al., Fetal myocardial deformation measured with two-dimensional speckle-tracking echocardiography: longitudinal prospective cohort study of 124 healthy fetuses. Ultrasound Obstet Gynecol. 2022 May;59(5):651–659. doi: 10.1002/uog.24781 Epub 2022 Apr 6. ; PMCID: PMC9321172. 34558747 PMC9321172

[18] Nichting TJ, Frenken MWE, van der Woude DAA, van Oostrum NHM, de Vet CM, van Willigen BG et al., Non-invasive fetal electrocardiography, electrohysterography and speckle-tracking echocardiography in the second trimester: study protocol of a longitudinal prospective cohort study (BEATS-study). BMC Pregnancy Childbirth. 2021 Nov 25;21(1):791. doi: 10.1186/s12884-021-04265-8 ; PMCID: PMC8613985. 34823483 PMC8613985

[19] Varma DR. Managing DICOM images: Tips and tricks for the radiologist. Indian J Radiol Imaging. 2012 Jan;22(1):4–13. doi: 10.4103/0971-3026.95396 ; PMCID: PMC3354356. 22623808 PMC3354356

[20] Nichting TJ, de Vet CM, van der Ven M, van der Woude DAA, van Sloun RJG, Oei SG et al., Angle Independency of Fetal Speckle-Tracking Echocardiography: A Commentary Letter. J Am Soc Echocardiogr. 2022 Jul;35(7):783–785. doi: 10.1016/j.echo.2022.02.013 Epub 2022 Mar 8. . 35271991

[21] Cheng S, Larson MG, McCabe EL, Osypiuk E, Lehman BT, Stanchev P et al. Reproducibility of speckle-tracking-based strain measures of left ventricular function in a community-based study. J Am Soc Echocardiogr 2013;26:1258–1266.e2. doi: 10.1016/j.echo.2013.07.002 23953701 PMC3812381

[22] DeVore GR, Satou G, Sklansky M. Comparing the Non-Quiver and Quiver Techniques for Identification of the Endocardial Borders Used for Speckle-Tracking Analysis of the Ventricles of the Fetal Heart. J Ultrasound Med. 2021 Sep;40(9):1955–1961. doi: 10.1002/jum.15561 Epub 2020 Nov 11. . 33174649

[23] Cocks K, Torgerson DJ. Sample size calculations for pilot randomized trials: a confidence interval approach. Journal of Clinical Epidemiology. 2013;66(2):197–201. 23195919 10.1016/j.jclinepi.2012.09.002

[24] Hazra A. Using the confidence interval confidently. J Thorac Dis. 2017 Oct;9(10):4125–4130. doi: 10.21037/jtd.2017.09.14 ; PMCID: PMC5723800. 29268424 PMC5723800

[25] Bobak CA, Barr PJ, O’Malley AJ. Estimation of an inter-operator intra-class correlation coefficient that overcomes common assumption violations in the assessment of health measurement scales. BMC Med Res Methodol. 2018 Sep 12;18(1):93. doi: 10.1186/s12874-018-0550-6 ; PMCID: PMC6134634. 30208858 PMC6134634

[26] Markkanen HK, Pihkala JI, Salminen JT, Saarinen MM, Hornberger LK, Ojala TH. Prenatal diagnosis improves the postnatal cardiac function in a population-based cohort of infants with hypoplastic left heart syndrome. J Am Soc Echocardiogr 2013;26:1073–9. doi: 10.1016/j.echo.2013.05.005 23891125

[27] Mahle WT, Clancy RR, McGaurn SP, Goin JE, Clark BJ. Impact of prenatal diagnosis on survival and early neurologic morbidity in neonates with the hypoplastic left heart syndrome. Pediatric 2001;107:1277–82. doi: 10.1542/peds.107.6.1277 11389243

[28] Enzensberger C, Achterberg F, Degenhardt J, Wolter A, Graupner O, Herrmann J et al., Feasibility and Reproducibility of Two-Dimensional Wall Motion Tracking (WMT) in Fetal Echocardiography. Ultrasound Int open. 2017 Feb 1;3(1):E26–33. doi: 10.1055/s-0042-124501 28210715 PMC5301653

[29] Erickson C, Levy P, Craft M, Li L, Danford D, Kutty S. Maturational patterns in right ventricular strain mechanics from the fetus to the young infant. Early Hum Dev. 2019 Feb 1;129:23–32. doi: 10.1016/j.earlhumdev.2018.12.015 30616038

[30] Kapusta L, Mainzer G, Weiner Z, Deutsch L, Khoury A, Haddad S et al., Second trimester ultrasound: reference values for two-dimensional speckle tracking-derived longitudinal strain, strain rate and time to peak deformation of the fetal heart. J Am Soc Echocardiogr. 2012 Dec;25(12):1333–41. doi: 10.1016/j.echo.2012.09.011 23200418

[31] Kim S, Miyakoshi K, Kadohira I, Tanaka M, Minegishi K, Matsumoto T, et al., Comparison of the right and left ventricular performance during the fetal development using velocity vector imaging. Early Hum Dev. 2013 Sep;89(9):675–81. doi: 10.1016/j.earlhumdev.2013.04.015 23707047

[32] Huntley ES, Hernandez-Andrade E, Soto E, DeVore G, Sibai BM. Novel Speckle Tracking Analysis Showed Excellent Reproducibility for Size and Shape of the Fetal Heart and Good Reproducibility for Strain and Fractional Shortening. Fetal Diagn Ther. 2021;48(7):541–550. doi: 10.1159/000517625 Epub 2021 Aug 25. . 34515112

[33] Yamada A, Luis SA, Sathianathan D, Khandheria BK, Cafaro J, Hamilton-Craig CR et al. Reproducibility of regional and global longitudinal strains derived from two-dimensional speckle-tracking and Doppler tissue imaging between expert and novice readers during quantitative dobutamine stress echocardiography. J Am Soc Echocardiogr 2014;27:880–7. doi: 10.1016/j.echo.2014.04.016 24891261

[34] Crispi F, Sepulveda-Swatson E, Cruz-Lemini M, Rojas-Benavente J, Garcia-Posada R, Dominguez JM, et al. Feasibility and reproducibility of a standard protocol for 2D speckle tracking and tissue Doppler-based strain and strain rate analysis of the fetal heart. Fetal Diagn Ther. 2012;32(1–2):96–108. doi: 10.1159/000337329 Epub 2012 Jun 19. . 22722425

[35] Ta-Shma A, Perles Z, Gavri S, Golender J, Tarshansky S, Shlichter C, et al. Analysis of segmental and global function of the fetal heart using novel automatic functional imaging. J Am Soc Echocardiogr. 2008 Feb;21(2):146–50. doi: 10.1016/j.echo.2007.05.007 Epub 2007 Jul 12. . 17628416

[36] Faber JW, Hagoort J, Moorman AFM, Christoffels VM, Jensen B. Quantified growth of the human embryonic heart. Biol Open. 2021 Feb 10;10(2):bio057059. doi: 10.1242/bio.057059 ; PMCID: PMC7888713. 33495211 PMC7888713

[37] Donald School Journal of Ultrasound in Obstetrics and Gynecology (2021): 10.5005/jp-journals-10009-1707

[38] Schneider U, Bode F, Schmidt A, Nowack S, Rudolph A, Dölker EM, et al., Developmental milestones of the autonomic nervous system revealed via longitudinal monitoring of fetal heart rate variability. PLoS One. 2018 Jul 17;13(7):e0200799. doi: 10.1371/journal.pone.0200799 Erratum in: PLoS One. 2018 Aug 14;13(8):e0202611. PMCID: PMC6049949. 30016343 PMC6049949

[39] Sivesgaard K, Christensen SD, Nygaard H, Hasenkam JM, Sloth E. Speckle Tracking Ultrasound Is Independent of Insonation Angle and Gain: An In Vitro Investigation of Agreement with Sonomicrometry. *J Am Soc Echocardiogr*. 2009;22(7):852–858. doi: 10.1016/j.echo.2009.04.028 19515531

[40] Nichting TJ, de Vet CM, van der Ven M, van der Woude DAA, Regis M, van Sloun RJG et al., The impact of angles of insonation on left and right ventricular global longitudinal strain estimation in fetal speckle tracking echocardiography. PLoS One. 2023 Jul 12;18(7):e0287003. doi: 10.1371/journal.pone.0287003 ; PMCID: PMC10337891. 37437044 PMC10337891

